# Ebola and slum dwellers: Community engagement and epidemic response strategies in urban Sierra Leone

**DOI:** 10.1016/j.heliyon.2023.e17425

**Published:** 2023-06-28

**Authors:** Zuzana Hrdličková, Joseph Mustapha Macarthy, Abu Conteh, S. Harris Ali, Victoria Blango, Alpha Sesay

**Affiliations:** a Sierra Leone Urban Research Centre (SLURC), Sierra Leone; b Njala University (NU) and Sierra Leone Urban Research Centre (SLURC), Sierra Leone; c York University (YU), Canada; d Njala University (NU), Sierra Leone

**Keywords:** Ebola, Epidemics, West Africa, Community engagement, Informal settlements, Slums

## Abstract

The Ebola epidemic in West Africa (2013–2016) was a learning process for all – the population, health experts and practitioners, as well as government structures. Learning occurred in all stages of the response, from the initial lack of clarity and denial of Ebola's existence that contributed to public confusion; to the eventual acceptance of the existence of the Ebola threat whereupon fear and stigmatization reigned; to the later stages in which community engagement and ownership of the response arose. In this paper we describe how two urban poor communities in informal settlements in the Western Area of Sierra Leone responded to Ebola Virus Disease and how they deployed efficient strategies like the development and implementation of by-laws for monitoring and surveillance, thus helping to curb the epidemic. For future public health emergencies, we recommend that community engagement be pursued earlier and that efforts are made to ensure two-way knowledge exchange between responders and community stakeholders.

## Highlights

• The response to the Ebola epidemic in Sierra Leone progressed from public disconnect to high levels of community engagement.
• Urban slum communities in Sierra Leone are often highly organized and able to mobilize effectively.
• During the Ebola epidemic, the communities responded in three stages: 1) denial, 2) acceptance, and 3) self-mobilization.
• Public received information and direction well from traditional or trusted sources of authority within their communities.

## 1 Introduction

**Fig. 1:**
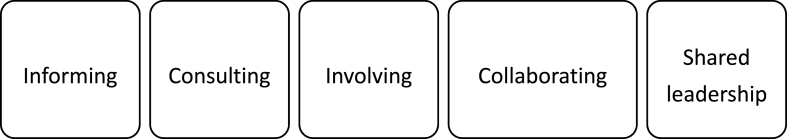
Continuum of Community Engagement presented in slides by World Health Organization, ‘Community Engagement’ slide 10, citing Victoria Government, Australia.

*Community engagement* has long been advocated and promoted within multiple research fields including development studies [1], disaster studies [2], and public health [3]. Some have termed it the community engagement turn [4], or see community engagement as part of contemporary zeitgeist [3]. Disaster studies make the point that community members are the first responders on site, as they are already *present at the affected location* long before the arrival of official responders. Involvement by community members is thus key to successful disaster impact mitigation [2]. However, some rightfully caution not to place all responsibility for disaster mitigation on communities, as risks are sometimes inadvertently produced by actors outside the affected communities [5]. Strategies of community engagement, therefore, must strike the right balance between local involvement and relevant external support. Such strategies may be seen as acts of *knowledge sharing* in which authorities share general knowledge of the risk and local communities share locally specific geographical, social, and cultural knowledge. Community engagement also plays a key role in public health emergency response for the same reason, as it is the communities who are directly affected. Community engagement may include a range of possible activities on the part of government such as “informing”, “consulting”, “involving”, “collaborating” and finally “shared leadership” (see Fig. 1).

Yet, during emergencies of any kind, a complaint is often voiced that communities are not meaningfully engaged by governments [[2], [3], [4]]. The failure of authorities to engage communities does not come as a surprise. As the term “authority” itself implies, government officials’ general aim is to execute government orders that govern or otherwise control populations, not to understand them, take their viewpoint or work with them. It is based on the “modern” conventional view held by governing officials that the public lacks knowledge or are otherwise non-experts - a perception that is well documented in science and technology studies [3]. The command-and-control dimension of governance is thus contrary to the strategy of involving and/or collaborating with communities. However, this conventional governance approach fails during public emergencies when behaviour change of the public is required for the crisis to end. It usually takes time for governments to realize the need to engage communities and switch from an authoritarian to a collaborative mode. These transitional phases tend to be messy and chaotic, when initially communities are often left to navigate the realities of the emergency with messages, guidance or orders that do not match the situation they are in.

In this paper, we provide an example of such transitional phase from sole command-and-control response to co-existence with collaborative community engagement in two urban poor communities in Western Sierra Leone. We provide a chronological account of how the 2013–2016 West African Ebola Virus Disease (EVD) epidemic evolved along with an overview of official response measures. We describe the community reaction *as a gradual and evolving process* that started with lack of clarity often accompanied by denial that led to the fear and stigmatization of Ebola survivors, and eventually leading to community engagement and ownership of the response. We proceed to describe the type of collaboration that progressively took place between the government-led response mechanism and communities through knowledge exchange, involvement of community leaders, community-led surveillance, and bylaws, leading to improved and trusted communication and sensitization. We suggest that internal community support groups and local justice system were key stones that helped get the epidemic under control. We join the growing number of researchers (e.g. refs. [[6], [7], [8], [9], [10]], arguing that communities should be included in epidemic preparedness and response as early as possible. While most literature on community engagement during Ebola focuses on rural communities, we argue also for meaningful inclusion of urban informal settlement communities, as they have efficient governance structures capable of significant grassroot impact. We also look at different conceptualizations of “meaningful” community engagement.

### 1.1 Ebola emerges in Sierra Leone

The 2013–2016 West Africa Ebola virus outbreak in Guinea, Liberia and Sierra Leone was the most severe outbreak of the disease to date, resulting in 11,325 deaths [7]. All three countries rank among the least developed and have some of the weakest health systems in the world with inadequate financing, workforce, governance, infrastructure, and service delivery [11]. In Sierra Leone at the time, there were 2.2 health workers per 10,000 population and approximately 4 hospital beds per 10,000 population (ibid). Between 2014 and 2016, the epidemic claimed 3956 Sierra Leonean lives [9] and had devastating social and economic effects.^2^

Walsh and Johnson [9] provided a detailed account of how Ebola emerged in the country. The virus was first confirmed in Sierra Leone's eastern districts of Kailahun and Kenema in May 2014, increasingly making its way through the country affecting towns like Bo, Moyamba and Port Loko. In July the situation was becoming serious, with Ebola spreading to the capital Freetown and rising numbers of fatalities, including among health workers. The passing of Dr. Umar Khan - the only Sierra Leonean virologist – was a wake-up call that unsettled the entire country, including political elites. The response mechanism at the time had numerous faults: All Ebola positive cases had to be transferred to the eastern treatment centres in Kenema and Kailahun, which often entailed an arduous drive of several hours. Government messages were not believed and conspiracy theories were rife, leading to riots in hospitals in Kenema and Freetown. According to Ross, it took some time for the government-led response to settle its inefficiencies, political rifts and mature [12]. The national government only started mobilizing their response in July by initially setting up the Emergency Operation Centre (EOC) based at the Ministry of Health, as well as the Task Force on Ebola, declaring a State of Public Emergency (30th July 2014) and quarantining Kenema and Kailahun districts. In August 2014, international pledges to support the response were made and in September and October 2014 Sierra Leonean authorities stepped up their efforts by establishing more treatment units closer to the capital and changing the response strategy by forming the National Ebola Response Centre (NERC), which was supported by the military in terms of coordination and logistics, and its district counterparts (DERCs). The epidemic situation in the first hit regions in the east was beginning to improve in the first week of October. It was recognized that this improvement was heavily facilitated by direct involvement of communities who had changed their behaviours. By this point the response authorities also realized that the situation had little chance of improvement without the population in other parts of the country willingly and consistently changing their behaviour. This required moving away from the pure command-and-control approach toward consideration of the experiences and needs of the population. In this spirit, the president and DERC officials started engaging local leaders in the districts to support Ebola response more effectively. International organizations started arriving while the epidemic was still spreading rapidly in other parts of the country, especially in Freetown, its surrounding rural areas, and in the northern districts of Port Loko and Bombali, reaching 5000 sick on 17th November. Finally, December 2014, saw the opening of most new (state- and internationally-led) laboratories, Ebola Treatment Units (ETUs) and community care centres, providing enough beds, and laboratory facilities. Throughout 2015 the numbers of sick were declining thanks to better sensitization, community engagement and contact tracing. The epidemic was declared over on 17 March 2016^3^ [13].

### 1.2 Initial weak response and lack of community engagement

Walsh and Johnson [9] assessed the reasons behind the weak response seen during the initial period of the epidemic between March and July of 2014. Looking beyond the fact that Sierra Leone had one of the weakest health systems in the world, they suggest that internationally, nationally and at the local level, the epidemic was often not taken seriously enough. Apart from Médecins Sans Frontières (MSF) who had been raising alarm, nobody seemed to be able to picture the potential impact of Ebola in West Africa. The government and the World Health Organization (WHO) adopted an ‘anti-alarmist’ approach focused more on calming the public rather than on preparing for the worst-case scenario. Communities often doubted Ebola existed, and some global Ebola experts at the time believed the virus would not spread to cities ([9]; 53), failing to consider the high level of mobility characteristic for the region, which was very different from the isolated communities affected by the previous Ebola outbreaks [8]. Opportunities to adequately prepare the health facilities and workforce for their first Ebola cases were missed due to inappropriate training formats, erroneous selection of trainees,^4^ as well as failure to deliver vital supplies of PPE kits and chlorine. The funding regimes of international donor organizations were inflexible and unable to provide the small funds needed for quick set up of Ebola isolation units in local hospitals [9]. Once Ebola appeared in Sierra Leone, health workers were not equipped to undertake their duties safely, and in the end, 221 died in the epidemic [14]. Moreover, it was documented that those in charge on local levels often provided inaccurate reports to their superiors, making it appear as if the situation was under control. As a result, in June 2014, the Ministry of Health in Freetown was still painting a positive picture about the Ebola response, while hospitals in Kailahun and Kenema were already seriously overstretched. Some saw Ebola as an opportunity for enrichment as a large proportion of response funds had not been properly accounted for. There was lack of leadership, and coordination was poor and often hampered by politics [9,12]. While most of these issues were gradually addressed and improved in late 2014 and through 2015, the biggest shortcoming of the response was the inadequate engagement of communities from the beginning of the epidemic.

*“Only the communities of Sierra Leone were ever going to be able to bring the Ebola epidemic under control. Only they could avoid unsafe practices. It was irrelevant how many ETUs we built if people didn't come to them, or how strong the safe burial system was if people didn't report deaths. Without communities pulling in the same direction as the response, it would never be successful.”* ([9]; 340)

In the initial stage of the epidemic, authorities were in pure command-and-control mode and had no appetite for listening to community views. Therefore, for most of 2014 communities were “engaged” in the form of one-way communication, which advised people to avoid bushmeat (whilst many affected had not eaten bushmeat), informed the public about symptoms of Ebola, and provided a helpline number. There was no listening and no real consideration of communities experiences and needs. There was no advice on how to safely care for the sick at home. The initial insistence of experts that home-care should be discouraged was shockingly disconnected from realities on the ground with inaccessible roads and scarce mobile coverage being the norm in many parts of Sierra Leone. Compounded with long distances to ETUs, families of the sick were left unsupported in chaotic and scary situations [8]. The lack of respect and empathy translated into insensitive measures (e.g., burial measures that did not consider local norms and traditions) that were often unacceptable in light of the cultural norms of the survivors [9,[15], [16], [17]]. The main paradigm of the official response was “containment over care” [18].

In other words, although communities were the first responders to the Ebola epidemic, they were poorly equipped with messages and orders that did not match the situation they were in, and were largely left to navigate the realities of Ebola on their own. It was a disorienting time to say the least and different communities responded to Ebola outbreaks differently based on their own specific experiences, resources, and leadership [8]. In this light, Richards suggests that the unfolding response to Ebola in West Africa be viewed as a “concatenation of local dissimilar events” [19]. The impact of the outbreak appeared to be worse in communities with limited economic opportunities and poor access to healthcare [20,21]. The transmission vulnerability was especially high in informal settlements because of intersecting factors such as density, household and social structures, mobility, livelihood imperatives, ventilation, access to water, toilets and waste disposal [22].

## 2 Materials and methods

### 2.1 Two urban poor settlements in Western Area

**Fig. 2:**
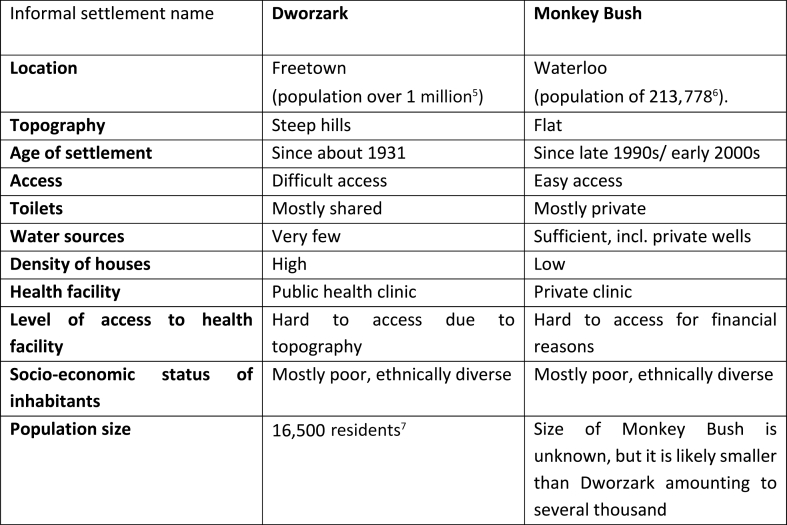
Characteristics of the researched settlements.

In this paper we share findings of our field study and show how two urban poor communities with different characteristics in the Western Area of Sierra Leone learned to respond to the Ebola epidemic. These two locations had been among the ‘‘hotspots’’ recording high rates of daily Ebola infections between September and November 2014 [23]. Dworzark is an informal urban settlement established in the first half of the 20th century and situated on steep slopes in central Freetown, Sierra Leone's capital. The more recently established and flat settlement of Monkey Bush is a part of the peri-urban town of Waterloo, known to be a crossroad in the Western Area Rural district and a gateway between Freetown and the rest of the country. The population density of Dworzark is quite high and it appears congested and chaotic, compared to the relatively sparse population in Monkey Bush with easier access routes. The two communities also have different levels of access to water and sanitation services (see Fig. 2). Inhabitants of Dworzark commonly share toilets and have access to very few water sources, whereas people living in Monkey Bush mostly have private toilets and multiple (untreated) water sources, including private wells. Both communities experience similar social conditions of poverty and both have limited access to health facilities. Dworzark has its own public health centre, though it is located at the bottom of a hill, forcing sick hilltop residents to be carried on people's backs down steep footpaths. Monkey Bush has a private clinic, which is often too expensive for the residents to use. To seek healthcare, they need to travel several kilometres to a public facility in Waterloo.^5^^,^^6^^,^^7^

Thus, while both communities can be classified as “poor urban informal settlements” having difficult access to healthcare, they are also distinct from one another.

### 2.2 Research methods

Our research methods were reviewed and approved by the Sierra Leone Ethics and Scientific Review Committee (SLESRC, ICF #11a), and by the Human Participants Review Sub-Committee of the Office of Research Ethics at York University, Toronto (Certificate #: e2018-346). We confirm that the study complies with all regulations. We confirm that informed consent was obtained from all participants.

Our population sample included 102 informants (51 in each settlement), including diverse gender and age groups with all participants being 18 years old or over. All participants provided informed consent. Data was collected in May 2019, that is, four and a half years after the communities experienced the full brunt of the epidemic. Therefore, the results can be understood as an investigation into collective (and selective) memory-making processes, which help individuals and communities make sense of their experience and carry forward lessons learned. Our qualitative research approach included ten key informant interviews, sixty individual interviews, and four focus group discussions with people who were either affected by EVD or were involved in the government- or community-led response. Key interviewees consisted of community chiefs, local military/police commanders, heads of local health facilities, educational institutions, or the highest local governance actors within the locality. Individual interviews included Ebola survivors, relatives of the deceased, caregivers, ambulance drivers, chlorine sprayers, community health workers (CHWs), traditional birth attendants (TBAs), security officers, community informants, contact tracers, local politicians, representatives of community-based organizations (CBOs), and journalists. We conducted focus group discussions (FGDs) with 32 people in total – two FGDs in each community, one with the male and the other with female participants.^8^ Discussion participants had the same characteristics as those in the interviews. However, individuals were only allowed to contribute in one of the methods to enhance diversity. For example, a participant who took part in the individual interview would not be allowed to participate in the FGD or vice versa.

## 3 Results

### 3.1 The process of learning how to respond to Ebola: community response

Anthropologist Paul Richards [8] described the Ebola epidemic in West Africa as a process of the *gradual learning of all* – people, experts, and authorities. Communities as well as many health workers had no prior knowledge of Ebola before the outbreak in 2013. Ebola experts were faced with a different context from their previous experience in isolated forest communities, making their public messages incongruent with the realities on the ground.^9^ Health officials were underprepared, and had small capacity to deal with the epidemic until a clear chain of command was put in place. However, as time passed, all the actors learned and adapted. The national and community^10^ response steadily improved in late 2014 and through 2015 [8]. Also, as we shall see, our two study communities in Western Area developed their own ways to respond, thereby enhancing their resilience. The process in these two locations started with a lack of clarity accompanied by denial (stage 1), followed by fear and stigma of Ebola survivors (stage 2), to community engagement and ownership of the response (stage 3). (The timing indicated below applies to the general approach of state-level response authorities and to Monkey Bush and Dworzark. However, the first hit eastern areas experienced the process in a more accelerated pace and the timing does not apply to them.)

#### 3.1.1 Stage 1 - Chaos and denial (late March to late September 2014)

The first stage was characterized by an unclear situation, unreliable information, ineffective communication, and lack of involvement of community leaders. When Ebola first began to be mentioned by media and the authorities, the initial government-led Ebola sensitization was essentially one-way communication with messages focused on bushmeat consumption in situations where many affected people had not actually consumed bushmeat. The information did not make sense and there was no communication or guidance for community leaders. This led to many different reactions in communities depending on the knowledge and experience of their leaders [8]. In the informal settlements of Dworzark and Monkey Bush, many people ignored health warnings. Ebola arrived in both communities via individuals who did not follow public health recommendations – one having had contact with a quarantined girlfriend (Dworzark) and another attending a funeral (Monkey Bush), both of whom hid and eventually infected their families. The study participants stated that given their lack of knowledge, they were not prepared for such an outbreak and its scale of devastation.

*“My people lacked knowledge and understanding about the virus and as a result, the virus was brought into the community […] the lack of understanding led to the reluctance of people to abide by the precautions instituted by health practitioners”.* (A political representative at Dworzark)

*“Denial of the existence of the disease was the main factor that led to the spread because people were not obeying all of the instructions, this led to the spread.”* (An elder at Monkey Bush).

The denial and lack of adherence to the recommended rules was made worse by the living conditions in informal settlements. The population density is especially high in Dworzark.

*“The way houses are clustered in Dworzark is a significant reason why the disease spread in the community. In this community, people depend on each other for a number of things, especially cooking items. You can see people moving from their houses to borrow cooking items and utensils from their neighbours. It has been a close-knit community such that people almost always drink water from the same cup or the same packet being shared by different people.” (*Dworzark, MP)

*“Congestion was also another reason the disease spread in the community. For instance, in a room and parlor [apartment with living room and bedroom], you can have as many as six family members who exchange sweat. This also contributed to spreading the disease”* (Head of Chiefs at Dworzark)

Denial of EVD contributed to rapid spread of the infection in both communities. In her commentary on COVID 19, Annie Wilkinson pointed out that communities in urban informal settlements live with constant risk alongside infectious diseases and the public health authorities usually do not intervene. It is therefore no surprise that informal community residents tend to view public health messages with suspicion and to not adhere to the guidance provided^11^ [22]. This point holds also for the case of Ebola.

#### 3.1.2 Stage 2 - Acceptance and fear (October 2014 to March 2015)

While the first large scale realization and acceptance of the Ebola threat by the authorities and the public in the eastern regions of the country were prompted by the death of virologist Dr. Umar Sheikh Khan at the end of July 2014, in the western part of Sierra Leone the threat of Ebola began to be recognized by communities only with increasing infection levels in October 2014. The severity of the situation was also underscored by the concurrent change in the official response strategy, when the highly respected Sierra Leonean military became heavily involved in the response as NERC [9]. At the same time community structures began to be considered and community leaders began to be gradually engaged, for example by the President. It was observed by our researchers that the existence of Ebola was more rapidly recognized in Dworzark rather than in Monkey Bush. However, there were still unclarities about the mode of transmission, and how one could protect themselves from Ebola. People still did not receive information from sources they trusted. There was deep-set fear of Ebola and survivors, and victims’ families were stigmatized,^12^ avoided and often left unsupported. Also the response workers based in our two communities (health workers, contact tracers or ambulance drivers) experienced ostracization and avoidance despite their high standing in the community.

*“Because I was a contact tracer, my family distanced themselves from me. Even my friends were never comfortable siting close to me. My wife was afraid to come close to me because she feared I would get her infected”* (Contact tracer in Dworzark)

*“Whenever I went out to sensitize people on what the government had instructed, I would immediately take my bath when I returned home, and all family members had to sleep separately instead of sharing the same bed. It was really not easy as all those relationships and interactions stopped; we did not shake people’s hands and our movements were restricted”* (A community leader at Monkey Bush)

In a situation when communities did not have reliable information from sources they trusted about how Ebola was transmitted, avoidance of contact altogether made sense. Ebola-related stigma in Sierra Leone has developed from the fear of the unknown and the way it was enacted has changed during the course of the epidemic.

#### 3.1.3 Stage 3 - Communities take ownership of the response (May 2015 to March 2016)

As the public health crisis evolved, the Ebola response authorities recognized that community engagement was key to the successful management of the epidemic. Unless communities and individuals changed their behaviours, Ebola could not be stopped. Therefore, communities had to become part of the response. However, the process did not happen in linear stages mentioned earlier (informing, consulting, involving, collaborating, shared leadership) but rather the stages unfolded in parallel. Crucially, this included better information sharing about Ebola with community leaders and improved public sensitization. As a result, communities started taking their own action, and undoing some of the fear characteristic of the previous stage and changing the way people reacted to stigma. Such a change in orientation included setting up of community bylaws and surveillance. Solidarity within communities manifested through various support groups, collaborative and individual action.

### 3.2 Factors associated with community ownership of response

#### 3.2.1 Improved information sharing through involvement of community leaders

In rural Sierra Leone, people come under the authority of chiefs and nothing happens without their approval. In urban slums, chiefs^13^ do not enjoy the same level of power and respect, as they are often seen as out of touch with modern life [24]. However, they still have power to mobilize the community by working in conjunction with the leaders of numerous local associations based in the slums, including secret societies, youth groups, women groups, sport associations, community development committees, and religious groups. These different actors can deploy effective measures if they are equipped with the right type of information. Basic training workshops about Ebola for key figures and community members provided them with the accurate knowledge and the skills needed to help them engage with their communities efficiently. In this way, a variety of community leaders including chiefs, elders and association leaders mobilized residents, and raised awareness. It was recognized that soft communication skills were required to manage the epidemic. One of the health responders noted:

*“We initially thought the disease could be taken care of by only health personnel but when it struck, we realized the intervention of non-health personnel would also be vital. It was only when councillors, chiefs and other local stakeholders were involved in the response team that we were able to develop effective response mechanisms”*.

As the government began to repeatedly engage community leaders, Ebola began to be better understood on the community level and this contributed to an increase in mutual trust. Community leaders were viewed as reliable sources of information. And the effectiveness of response increased further when information began to be disseminated to residents in the study settlements by their own community leaders and youth and on posters and flyers about Ebola using local languages (Krio, Temne, and Mende). The improved understanding led to increased community ownership of the problem.

An Ebola survivor noted:

“This approach of community involvement in information dissemination was much better because we were able to understand the fight better through sensitization done by our own brothers and sisters. This also helped many people to believe the messages that were conveyed to them”

The authorities were aware of the lack of trust their initial mishandling of the epidemic caused and so they conducted several focused interventions, including large-scale house-to-house sensitization campaigns during Operation Western Area Surge^14^ and the two lockdowns in September 2014 and in March 2015. These initial state-led actions in 2014 were based on a command and control approach using the country's security forces (Police and Military) as the main agents for enforcement. However, also youth and other volunteers were trained on how to communicate information about Ebola and its mode of transmission and cascaded these messages to their peers and neighbours.

As explained by a community leader at Dworzark:

“We instituted many strategies that resulted in the rapid eradication of the EVD [Ebola Viral Disease] like the formation of community ward coordinators and community sensitizers. We set up a responsible team that went house to house to sensitize the people about the dangers of the EVD, what to do and what not to do”.

With community-led information dissemination, residents who had initially been resistant to frontline responders started accepting surveillance officers and contact tracers into their neighbourhoods and homes. Some even called the emergency responders when they realized that they were feeling sick. Households procured hand washing materials and placed them at their doorsteps. Sensitization significantly changed community attitudes and behaviours.

Moreover, other new developments, such as the establishment of new treatment centres in closer proximity to communities offering free care and food, and the increasing awareness that one could survive Ebola if treatment was sought early, also contributed to the change [8,9].^15^

#### 3.2.2 Community surveillance

Anthropologists have pointed out that government-led surveillance teams were not effective within many communities due to their disconnect from the local social fabric. Communities saw them as strangers, who by their very nature of being from outside were unable to understand the local context, norms or carry out all required social obligations associated with looking after the sick and the dead [8,15]. Government-led initiatives, such as the “wach u neiba” (“keep a watchful eye on your neighbour ”)^16^ neighbourhood watch – type scheme often fostered conflicts. This was the case especially in Monkey Bush. However, in places where community leaders took charge and instituted bylaws to limit the mobility of people, such as in the first hit communities in Kailahun, saw the epidemic being better managed. This approach was highly successful and it was recognized that only respected traditional authority were likely to be more successful in implementing restrictions to curb the epidemic. Therefore, after consultative meetings with government and health workers, community leaders in both Dworzark and Monkey Bush set up community surveillance task force teams consisting mainly of young community activists. Communities were much more accepting of these bylaws as they had been instituted by traditional authority. Knowing local geography, residents, their norms, and narratives enabled the teams to efficiently monitor quarantine homes and civilian movement. This allowed tracing of suspected cases that had until then avoided detection. A former contact tracer explained:

“We had a surveillance team that moved from house to house in search for sick people or dead bodies to avoid contact or illegal burial.… We had checkpoints all around the community (…) [with] thermometers that we used to check temperatures especially during the evening hours. What prompted us to form those checkpoints was when prevention of burials was enforced and people started smuggling dead bodies for burial.”

The community-led teams successfully found deceased and infected persons in individual households. They also traced quarantine escapees, bringing them back to continue their quarantine.

#### 3.2.3 Community justice system and bylaws

People often did not adhere to national government mobility restrictions [25,26]. Therefore, community leaders established and enforced bylaws to help reduce the spread of the disease. The bylaws varied from one community to another. In both of our study settings bylaws required homes to have Veronica buckets,^17^ soaps for hand washing and regular cleaning of resident compounds. Illegal burials and washing of dead bodies were banned. The bylaws also banned strangers (people usually not resident in the community) from entering the community,^18^ and commercial bike riders were not allowed to drive sick people to herbalists. A community leader at Dworzark explained:

“During the outbreak people were told not to gather, touch or wash dead bodies. I was responsible for implementing these bylaws that were passed by the authorities in this community”

This way communities were vigilant and aware of unnecessary movement.

Bylaws were also instituted against stigmatizing victims and survivors and if such instances were revealed, perpetrators would receive heavy fines or face time at the police station^19^ or court.

*“We organized a community meeting and as a community we threatened to shame and fine anyone provoking a survivor.”* (Town Chief at Dworzark)

*“Whoever was proved to have stigmatized a survivor was to pay a fine of five hundred thousand Leones (Le 500,000)*^20^*and if the person refused to pay we would mobilize as a community and take the matter to court”* (Former Councilor at Dworzark)

*“When the issue of “finger pointing” at people as [disease] carriers became rampant in the community, a [by]law to take the case to the chiefs was made. People were then constantly invited by the Headman to explain themselves why they have made such conclusion about their fellow community members. People stopped pointing fingers at others”* (Elder, a contact tracer at Monkey Bush).

Community leadership and bylaws thus contributed to partially diminishing the stigma experienced by survivors. There were no reports of people pushing back against the rules. It seems the bylaws were widely accepted because they came from a traditional respected source of authority.

#### 3.2.4 Collaborative action, external support and individual action

The strong ownership of the response by communities enhanced their ability to generate resources to support local actions. Despite resource challenges at the peak of the outbreak, communities enhanced their resilience by relying on strong social capital and networks. Many residents also volunteered to serve in government-run surveillance and sensitization teams.

Some local initiatives started even before the government’s official response, whilst others ran alongside it and individuals acted within their area of expertise. For example, in Monkey Bush, a private health practitioner had initiated radio talks to raise awareness about Ebola. Another health worker there aimed to ensure the safe use of chlorine by teaching people how to make the correct chlorine mixture^21^ and a pharmacist supplied medicines to quarantined people in the community:

“The Monkey Bush community was deprived of any form of medical facilities, when the Ebola started … I took some of my staff to see what little help we could render to the deprived people who were in quarantine, since I had a pharmacy. I supplied drugs like ORS [oral rehydration solutions] to children having frequent stool as well as adults”

At Dworzark, a community leader personally raised awareness about the use of hand gloves and demonstrated how to use them. He then distributed them to residents, especially those who had frequent contact with the public like motor bike riders and market sellers.

Community leaders and stakeholders also drew support from NGOs, the Red Cross and government agencies who supplied essential items such as gloves, handwashing buckets, soap and food. The non-governmental actors also provided crucial assistance removing dead bodies and the community perceived their actions as quicker and more flexible:

*“It was difficult on some days to have the government response team collect bodies or sick people. But for the Red Cross, they responded promptly whenever our brothers called them”* (Female Ebola survivor)

#### 3.2.5 Internal support groups

During the Ebola outbreak, many people lost their ability to earn livelihoods either because of restricted mobility during the lockdowns or by being placed in quarantine. The impact was often mitigated by local social support systems. The study participants described locally organized savings groups and credit schemes as critical for overcoming economic challenges and psychological trauma. Credit schemes helped those who wanted to start small businesses or meet family needs. Some of the loan arrangements used funds revolving among group members, while others involved individual money lending to friends and relatives.

*“Things became very difficult for us in this community. We had no other means of survival since our source of livelihood was put on hold. The only way some of us survived was by asking for loan from a woman who was offering loans to people. Though we had to return the money with interest, it was better than nothing.”* (Local herbalist)

Community, faith-based and not-for-profit organizations provided support to community residents, including psychosocial support to their congregation members and other people in need. They sent their young followers to visit quarantined homes to talk with the occupants and offer help.

*“The head of my church provided rice and other items like buckets and soap to give to the church congregation, this was happening in many other churches”* (Christian priest, Dworzark)

Whilst points 3.2.1 to 3.2.5 show the similar developments in the ways communities took ownership of the response, it needs to be said that there were also differences between Dworzark and Monkey Bush. Many residents in the more established (longer existing) urban community in Dworzark in central Freetown were more readily collaborating with the official response at the expense of their own safety within the community. Also, community-led surveillance teams in Dworzark were highly successful. A contributing factor to this better control of the situation was the topography of the settlement with Dworzark being on steep slopes, having only one entry point at the bottom of the hill. In general, the infection in Dworzark was under much better control despite having worse sanitary conditions than Monkey Bush. On the other hand, the peri-urban Monkey Bush in Waterloo was much more open to the surrounding landscape. People could easily enter and leave the settlement in this much more accessible terrain. The resistance against government measures was strong in Monkey Bush and community led surveillance was established here later than in Dworzark.

## 4 Discussion: gradual development of meaningful community engagement

In this paper, we have described how between 2014 and 2016 Ebola gradually affected the whole of Sierra Leone. Its containment required improved functioning of local response, assistance from international actors, and better community engagement. The weaknesses of the initial stages of the response included lack of knowledge and failure to involve communities. Health experts knowledgeable about Ebola did not know how the epidemic would evolve within the local context. Local leaders had local knowledge but limited awareness of the disease and how it was transmitted. Government agencies did not act with sufficient urgency and did not communicate well enough with the public. Most publications describe situations in rural communities. Our paper on the other hand, described gradual emergence of community action in two informal urban communities - Monkey Bush (Waterloo) and Dworzark (Freetown). The epidemic evolved differently in various communities depending on the experience of their leadership and so Ebola can be viewed as a concatenation of dissimilar events [19]. That is also the case of our two informal settlements.

**Fig. 3:**
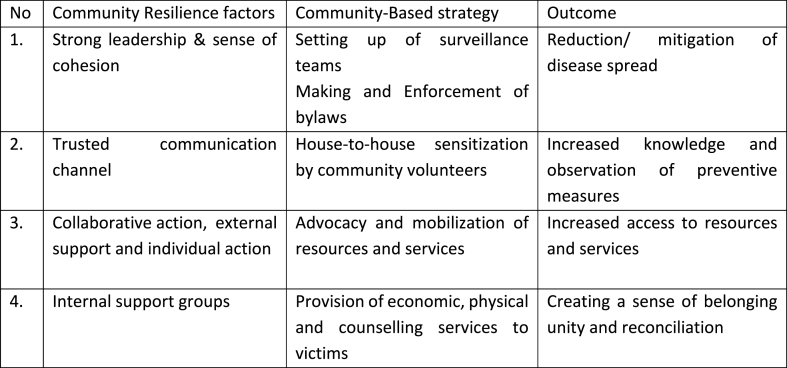
Community Resilience Factors, Strategies and their Outcomes.

Source: Survey Data.

In popular imagination, urban slums are seen as places of chaos. However, they are in fact highly organized through chiefs, associations, and other community actors. We depicted the three stages of response within these communities as follows: (1) chaos and denial (2) acceptance and fear, and (3) communities taking ownership of the response. In the initial absence of reliable information (1 and 2), where the nature of disease and the means of transmission were not exactly understood by the wider group, physical separation became a meaningful instrument to avoid contagion. This was in part facilitated by fear and stigma [27,28] in the first two stages. However, as noted by the Ebola Response Anthropology Platform (2014), physical separation or distancing did not need to come with negative labelling, and communities could have the ability to develop their own culturally appropriate protocols to avoid transmission. In this sense, when communities took ownership of the response, they started the process of undoing fear and stigma. Fig. 3 shows how different community resilience factors, such as strong leadership, trusted communication channels, collaborative action and internal support groups and associated strategies led to positive outcomes which contributed to improved control of Ebola epidemic.

The gradual community engagement started with government informing and involving local leaders, to collaborating with them, and finally local leaders taking charge, translating health precautions into action, and instituting and enforcing their own bylaws, mandating local residents to conduct community surveillance, and sensitize other residents. The success of community response relied on their own community justice system, social support system and collaborative action drawing on external and internal support. Community leadership took a stance against open stigmatization through introducing bylaws. Thanks to their action, the stigma endured by survivors has significantly decreased although not disappeared.

Only creating a joint platform where dialogue between those who held information about the disease, those who held information about local customs and context (often through assistance of anthropologists), and those with social capital and power to mobilize people could result in a successful fight against the disease. The subsequent stages of the epidemic thus saw high levels of community engagement facilitated by local leaders, authorities, NGOs, and social scientists^22^ who assisted in building locally-appropriate interventions. At the same time, the situation also improved thanks to Sierra Leone military being firmly in charge of the national response (with support of international actors). We therefore argue that meaningful community engagement *can* and should exist alongside improved command and control system which ensures efficient operational capacity of the official response.

There is much discussion about what “meaningful” and “impactful” community engagement may mean. Sometimes the term community engagement can be tokenized as a cure for all inequalities when in fact it can perpetuate them [5]. In the most basic human terms, community engagement requires mutual respect and willingness to listen and compromise [9]. It also means aiming to be prepared for messiness [3], thorough community, kinship, and cultural mapping [7] and inclusion of diverse groups [16]. Attempting community engagement is always better than avoiding it altogether [2]. Since communities’ involvement is fundamental to establish control of disease outbreaks, Anoko et al. call for a paradigm shift from predominantly biomedical approach to outbreak response to one that balances biomedical and social science approaches. In their view, health actors, community leaders and communities should jointly co-construct responses which are acceptable and feasible and foster commitment of affected communities. They share practical tips how do achieve this process [6].^23^

## 5 Conclusion

We have found that there is adequate agency within urban poor communities to improve health outcomes. These communities have effective leadership structures driving appropriate responses, deploying effective surveillance and communications, and utilizing indigenous social support systems. During the EVD epidemic the Sierra Leonean government and health workers were able to build on these strengths overtime to increase community acceptance for an effective response. These learnings demonstrate the need for community inclusion in emergency health planning interventions, which could be leveraged upon by policy processes.

## Author contribution statement

Zuzana Hrdlickova; Abu Conteh: Analyzed and interpreted the data; Wrote the paper.

Joseph Mustapha Macarthy; S. Harris Ali: Conceived and designed the experiments; Analyzed and interpreted the data; Wrote the paper.

Victoria Blango; Alpha Sesay: Performed the experiments; Analyzed and interpreted the data.

## Data availability statement

Data will be made available on request.

## Declaration of competing interest

The authors declare that they have no known competing financial interests or personal relationships that could have appeared to influence the work reported in this paper.

## Footnotes

2 Nearly 70% of economically active Sierra Leoneans reported that their revenue decreased during the epidemic [29]; p.2).

3 In fact, the epidemic was declared over first on 7th November 2015. However, new cases appeared in January 2016 in Tonkolili district and Sierra Leone saw the official end of the epidemic for the second and final time, on 17 March 2016.

4 Walsh and Johnson describe the persistent per diem culture, which meant that people would be motivated to attend trainings by financial payments. As a result, attending trainings was highly sought after even among people to whom the content of the training was irrelevant. For this reason, Ebola preparatory trainings were neither attended by frontline health staff who would in reality encounter first Ebola cases, nor by people who would be in a position to share their learning and adapt hospital management and safety processes.

5 see FCC, Transform Freetown: an Overview. 2019-2022, Freetown City Council, 2019 [30].

6 Statistics Sierra Leone, 2015 Population and Housing Census Summary of Final Results; Planning a Better Future (2015) [31].

7 ‘Change by Design. Dwozark Community Action Area Plan.’ 2018 [32].

8 Each focus group consisted of eight participants with a total of sixteen per community (eight males, eight females).

9 The context changed from isolated forest communities of DRC to the interconnected West Africa with strong rural-urban links and frequent travel.

10 The worst affected districts improved already before improved national and international intervention thanks to awareness campaigns and communities changing their behaviour.

11 While Wilkinson applied this point to Covid 19 situation, this statement is applicable to the context of Ebola as well.

12 According to the Ebola Response Anthropology Platform (2014), stigma is a consequence of a number of processes “that brand someone as different in ways that result in discrimination, loss of status and social exclusion”.

13 In Sierra Leone, chiefs were actively involved to protect communities from outside intruders and government forces during the war [33]. In informal settlements, they are respected (usually male) figures who have resided in the community for many years and often own multiple properties in the settlement. Their function is for life.

14 December 2014 and January 2015.

15 According to Richards [8], treatment of Ebola became increasingly successful when similar procedures to treatment of cholera were introduced.

16 The authorities (Ministry of Health and later NERC) asked residents to give information on any suspected case within their neighborhoods to the contact tracers or by calling 117.

17 Water bucket with tap near the bottom for hand washing.

18 Traditionally in Sierra Leone, chiefs always have to be informed about arrival of a stranger, and their presence must be approved by them.

19 *“Sometimes, some people were reporting others who shouted at them saying “Please push Ebola is passing” [keep away from this Ebola afflicted person] to the police. However, the elders and the policy will intervene in the cases that came to the station to arbitrate but if unsuccessful, the case can be charged to court”* (police officer at Waterloo police station).

20 *Around 130 USD in 2016*.

21 *“Chlorine was causing a lot of problems in the community as many people who had it were not making the right mixtures; the mixture was very harsh … and it was causing harm to people. I therefore brought chlorine to the community and taught them how to make the correct mixture”*.

22 Set up in late 2014, the Ebola Response Anthropology Platform provided valuable resources and insights on how to work with communities, and set of recommendations has been published [[7], [10]].

23 Anoko et al. share the following 10 lessons from the West African Ebola for better community engagement during public health emergencies: 1. involve social scientists early in the response; 2. mobilize family leaders for surveillance, case detection, contact identification and follow-up and quarantine; 3. treat contacts with dignity and the empathy they deserve; 4. communicate laboratory results promptly; 5. care for the severely ill, while maintaining family connections; 6. prevent stigmatization of people and the families of those who recover; 7. recruit local staff in the response and involve local people to build response structures; 8. mobilize and involve resistant communities in the response to overcome dissent; 9. involve grass-roots leaders in the preparation and implementation of response measures; 10. mobilize media players, including social media networks [6].
